# Differential Strategies of Two Arbuscular Mycorrhizal Fungi Varieties in the Protection of *Lycium ruthenicum* under Saline–Alkaline Stress

**DOI:** 10.3390/jof10080554

**Published:** 2024-08-06

**Authors:** Xu Zheng, Ao Li, Ruining Nie, Chengxu Wu, Xinying Ji, Jiali Tang, Junpei Zhang

**Affiliations:** State Key Laboratory of Tree Genetics and Breeding, Key Laboratory of Tree Breeding and Cultivation of the State Forestry and Grassland Administration, Forestry Research Institute of China Academy of Forestry, State Forestry and Grassland Administration, Beijing 100091, China; woshizhengxu2022@163.com (X.Z.); a17637359516@163.com (A.L.); nieruining@163.com (R.N.); maoliwcx@163.com (C.W.); jixinying111@163.com (X.J.); tangjl202205@163.com (J.T.)

**Keywords:** black wolfberry, saline–alkaline stress, physiology and biochemistry, mycorrhizal fungi

## Abstract

To delve into the growth and physiological adaptations exhibited by the economically vital black wolfberry (*Lycium ruthenicum*) upon inoculation with arbuscular mycorrhizal fungi (AMF) under varying levels of saline–alkaline stress A series of pot experiments were conducted in a gradient saline–alkaline environment (0, 200, 400 mM NaCl: NaHCO_3_ = 1:1). One-year-old cuttings of black wolfberry, inoculated with two AMF species—Funneliformis mosseae (Fm) and Rhizophagus intraradices (Ri)—served as the experimental material, enabling a comprehensive analysis of seedling biomass, chlorophyll content, antioxidant enzyme activities, and other crucial physiological parameters. This study demonstrated that both Fm and Ri could form a symbiotic relationship with the root of *Lycium ruthenicum*. Notably, Fm inoculation significantly bolstered the growth of the underground parts, while exhibiting a remarkable capacity to scavenge reactive oxygen species (ROS), thereby effectively mitigating membrane oxidative damage induced by stress. Additionally, Fm promoted the accumulation of abscisic acid (ABA) in both leaves and roots, facilitating the exclusion of excess sodium ions from cells. Ri Inoculation primarily contributed to an enhancement in the chlorophyll b (Chlb) content, vital for sustaining photosynthesis processes. Furthermore, Ri’s ability to enhance phosphorus (P) absorption under stressful conditions ensured a steady influx of essential nutrients. These findings point to different strategies employed for Fm and Ri inoculation. To holistically assess the saline–alkaline tolerance of each treatment group, a membership function analysis was employed, ultimately ranking the salt tolerance as Fm > Ri > non-mycorrhizal (NM) control. This finding holds paramount importance for the screening of highly resilient *Lycium ruthenicum* strains and offers invaluable theoretical underpinnings and technical guidance for the remediation of saline–alkaline soils, fostering sustainable agricultural practices in challenging environments.

## 1. Introduction

Soil salinization, a pressing environmental concern exacerbated by climatic variations and topographical disparities, poses a formidable challenge. Saline–alkaline stress elicits synergistic impacts, precipitating physiological and metabolic imbalances within plants cultivated in saline soils. This phenomenon drastically elevates the levels of reactive oxygen species (ROS), instigating oxidative harm to vital biomolecules like membrane lipids and proteins, ultimately stifling plant growth and development [1]. Notably, the Qinghai region of China is predominantly characterized by salt–alkali soils, which harbor not only NaCl-dominated neutral salts but also NaHCO_3_-rich alkaline salts [2]. Combining NaCl and NaHCO_3_ in a 1:1 molar ratio more accurately simulates the comprehensive influence of diverse saline–alkaline components in the soil, enabling a nuanced evaluation of plant resilience to this stress.

In response to the deleterious effects of salinization, plants have gradually honed an array of adaptive mechanisms to effectively counteract salt–alkali stress. These encompass adaptive physiological adjustments, modulation of endogenous hormones, and preservation of ionic homeostasis [3]. Upon sensing salt–alkali stress signals, plants actively engage in osmotic regulation, leveraging organic solutes such as proline (Pro), soluble sugars (SS), soluble proteins (SP), and inorganic ions as crucial indicators of their osmotic adjustment prowess [4]. Moreover, when the delicate equilibrium between ROS production and scavenging is disrupted, pivotal antioxidant enzymes derived from mitochondria and chloroplasts, including superoxide dismutase (SOD) and peroxidase (POD), come into play, mitigating the oxidative stress induced by excessive ROS. Overexpression of SOD has been demonstrated to bolster growth traits and elevate resilience against salt stress in *Solanum nigrum* [5] and sweet potato [6], underscoring its pivotal role. Moreover, the intricate interplay of endogenous plant hormones is vital in modulating water homeostasis and maintaining cell membrane integrity amidst salt–alkali stress conditions [7]. Variations in the translocation and local synthesis of growth-promoting hormones, including gibberellin (GA3), indole-3-acetic acid (IAA), and zeatin riboside (ZR), elicit specific growth adaptations to saline–alkaline stress, further enhancing plant resilience [8].

Arbuscular mycorrhizal fungi (AMF) forge a mutually beneficial association with host plants, significantly optimizing their physiological status and fortifying them against a broad spectrum of environmental stressors [9]. In the case of peanuts [5], AMF symbiosis has been found to refine the root zone environment and physiological attributes, thereby escalating the plant’s tolerance to saline–alkaline. This symbiosis enhances the host’s capacity to assimilate vital nutrients like potassium and calcium, which in turn augments biomass accumulation across tissues and organs. This promotes the absorption of light energy under stress conditions, increases the content of photosynthetic pigments, and mitigates damage to the photosystem [10]. Furthermore, elevated levels of trace elements modulate the activity of crucial metal antioxidant enzymes, notably SOD, mitigating ion toxicity and safeguarding the normal architecture and functionality of plant cells [11]. AMF significantly enhances the osmotic adjustment capabilities of the host plant by stimulating the upregulation of pivotal genes engaged in the biosynthesis of various proline precursors, notably 1-pyrroline-5-carboxylic acid synthetase (P5CS) and glutamate dehydrogenase (GDH). Concurrently, it fosters an augmentation in photosynthetic proficiency, thereby satisfying the carbon requirements and effectively facilitating the accumulation of soluble sugars (SS) [12]. Additionally, AMF elicits the production of endogenous plant hormones as a defense mechanism against environmental stresses. By elevating the concentrations of indole-3-acetic acid (IAA) and gibberellins (GAs), it stimulates cell division and growth, while ABA (abscisic acid) synthesis collaborates synergistically with other hormones to mitigate the detrimental impacts of stressful conditions. Recent studies have demonstrated AMF colonization to alleviate growth retardation and oxidative stress in pepper [13], rice [11], and *Populus euphratica* [14]. Relevant studies have shown that Fm and Ri can be widely used as dominant fungi to regulate the salt tolerance of *Lycium ruthenicum* in the soil collected in Ningxia and Qinghai, the main planting areas, and have high ecological adaptability and application potential. At the same time, the two strains were easy to culture and inoculate into plant roots under experimental conditions, and have been confirmed in *Ligustrum vicaryi* [15], sour orange [16], and other plants.

Black wolfberry (*Lycum ruthenicum*), a resilient halophyte belonging to the Solanaceae family and *Lycium* genus, predominantly thrives in the arid landscapes of Qinghai, Ningxia, and Gansu provinces in northwestern China. Renowned for its robust tolerance to salinity, drought, and frigidity, black wolfberry emerges as an exemplary pioneer species, poised to rejuvenate soils and ecosystems [17]. Additionally, due to its abundant concentration of anthocyanins, polysaccharides, and amino acids, the plant exhibits health-promoting attributes, notably antioxidant and anti-hyperglycemic effects, thereby enhancing its suitability for application and advocacy in the arid landscapes of northwest China [3]. Previous studies have mainly focused on red wolfberry (*Lycum barbarum*), with relatively few studies on black wolfberry. Moreover, the existing studies mainly explore aspects such as photosynthesis under salt stress [18], leaf anatomical structure [19], and fruit quality [17]. Consequently, this study endeavors to fill this knowledge gap by examining the growth dynamics and physiological adjustments in black wolfberry subjected to saline–alkaline stress, facilitated by AMF inoculation. Through meticulous analysis of these changes, our objective is to elucidate the intricate physiological adaptation strategies employed by black wolfberry in saline–alkaline environments, ultimately furnishing a robust theoretical foundation for its cultivation and proliferation in the northwest region of China.

## 2. Materials and Methods

### 2.1. Experimental Design

**Figure 1:**
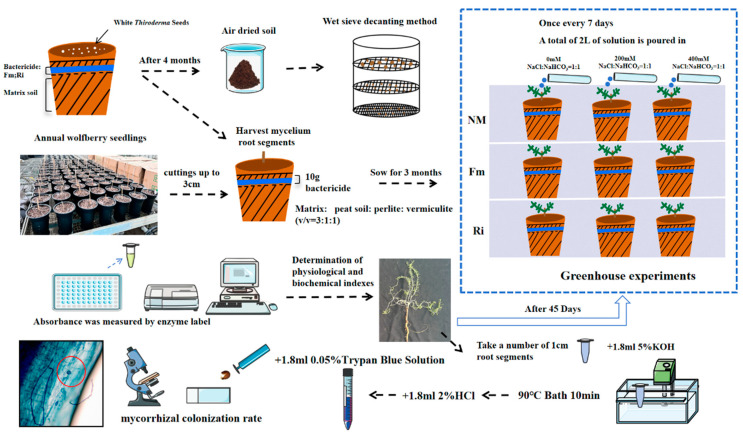
Experimental design.

The experiment was meticulously executed within the greenhouse facilities of the esteemed Chinese Academy of Forestry Sciences (40°0′10″ N, 116°14′38″ E). The experimental design, shown in Figure 1, comprises three integral segments: the cultivation of fungal species, the materials and treatment procedures, and the quantification of mycorrhizal colonization rates.

#### 2.1.1. Culture of Fungal Species

Funneliformis mosseae (Fm) and Rhizophagus intraradice (Ri) were sourced from the “Resource Conservation Center of Subtropical Arbuscular mycorrhizal AM Fungi”, Institute of Microbiology, Guangxi Academy of Agricultural Sciences. These mycorrhizae, enriched with spores, hyphae, root segments, and substrate, boasted an active spore count of 50 spores/g. To propagate the inoculum, white clover was sown in a meticulously formulated substrate blend of peat moss, coco peat, vermiculite, and perlite in a ratio of 5:2:2:1. The substrate was filled to 2/3 of the culture containers, followed by an even distribution of 15 g of mycorrhizal agent, and topped with another layer of the substrate mix. The ideal temperature range was maintained at 18–32 °C, with a relative humidity of 60–75%, ensuring optimal growth conditions. AM fungal spores were subsequently isolated from soil samples employing the refined wet sieve decantation technique [20], achieving a spore density of 20 spores/g. Upon harvest, the clover pots underwent a two-week drying period, with the aerial portions trimmed, and the entire culture (inclusive of clover root segments, hyphae, spores, and substrate) was carefully gathered, crushed using a short-handled ax, and stored in bags at a stable temperature of 25 °C.

#### 2.1.2. Material and Treatment

The cuttings of Qinghai black wolfberry, selected for uniformity in one-year-old growth and free from pests or diseases, were trimmed to a length of 4 cm and transplanted into pots of 25 cm height, featuring an upper diameter of 10 cm and a lower diameter of 11 cm. Each pot received a single cutting and was filled with 1000 g of a sterilized substrate blend comprising peat soil, vermiculite, and perlite in a ratio of 3:1:1 (*v*/*v*). The physical and chemical attributes of this tailored substrate, tailored specifically for optimal planting conditions, are detailed in Table 1.

Each pot was meticulously filled with 1000 g of thoroughly sterilized substrate, containing peat soil: vermiculite: perlite (3:1:1, *v*/*v*) and inoculum (20 g of inoculum/pot containing 150–200 spores) layered around the roots, followed by a cover of 0.5 kg of sterilized soil. Post-transplantation, the plants were nourished with a carefully calibrated low-phosphorus Hoagland nutrient solution, administered at a rate of 200 mL per pot every ten days. After approximately three months of growth, sufficient for the establishment of symbiotic relationships, the plants were subjected to saline–alkaline stress. NaCl and NaHCO_3_ were mixed with a molar ratio of 1:1, with three levels (0 mM, 200 mM, and 400 mM) of saline–alkaline solution categorized into NM, Fm, and Ri treatments, resulting in a total of 9 treatment groups. Each treatment was replicated three times biologically, with each replicate utilizing five strains. Prior to the treatment, a water control was implemented four days earlier to ensure uniform diffusion of the salt solution throughout the uniformly dried substrate. To maintain optimal hydration, each pot was watered with 300 mL of solution every 7 days, amounting to a total of 2 L over the course of the irrigation schedule. To capture excess water and nutrient solution, ensuring its prompt return to the pots, plastic trays were strategically placed beneath each pot. On the 45th day of stress exposure, the delicate roots and leaves of the seedlings were meticulously harvested and preserved in a −80 °C freezer for subsequent physiological index measurements.

#### 2.1.3. Mycorrhizal Colonization Rate Determination

For the assessment of mycorrhizal colonization, the roots of the wolfberry plants were harvested, dried with tissue paper, and cut into approximately 1 cm segments. These segments were then mixed, transferred to 2 mL centrifuge tubes, and subjected to a staining process using Trypan Blue solution (0.05% in lactoglycerol) [21]. This involved immersing the root segments in a 5% KOH solution at 90 °C for 10 min, followed by neutralization with a 2% HCl solution. Subsequently, 0.05% Trypan Blue dye was added and mixed thoroughly. For microscopic observation, 50% glycerin was added, enabling the visualization of mycorrhizal colonization under an optical microscope. Finally, the mycorrhizal infection rate was meticulously calculated.

Infection rate = (number of infected roots/number of detected roots) × 100% (1)

### 2.2. Experimental Methods

For each treatment, six seedlings exhibiting uniform growth patterns were meticulously selected. Both the aboveground and root portions were harvested, thoroughly rinsed with distilled water, and subsequently dried to determine the fresh weights: shoot fresh weight (SFW) for the aboveground portion and root fresh weight (RFW) for the root system. Additionally, tissue samples were enclosed in sealed envelopes, subjected to a rapid killing process at 105 °C for 15 min, and then dried at 80 °C until a constant weight was achieved. This enabled the determination of the dry weights: shoot dry weight (SDW) for the aboveground tissue and root dry weight (RDW) for the root tissue.

Chlorophyll and carotenoid content measurement: adopting the ethanol extraction method [22], the chlorophyll and carotenoid contents were accurately determined by measuring the absorbance at specific wavelengths: 665 nm, 649 nm, and 470 nm.

Chlorophyll a (Chla) = 13.95 × OD665 − 6.88 × OD649 (2)

Chlorophyll b (Chlb) = 24.96 × OD649 − 7.32 × OD665(3)

Carotenoids (Car) = (1000 × OD470 − 2.05 × Chla − 114.8Chlb)/245(4)

Total chlorophyll (TChl) = Chla + Chlb(5)

MDA, hydrogen peroxide (H_2_O_2_), and relative electrical conductivity (REC) analysis: MDA was determined using the thiobarbituric acid method [23]. H_2_O_2_ content was measured using the Hydrogen peroxide measuring kit (BC3595, Beijing, China). The relative electrical conductivity (REC) of the leaves was measured with an electrical conductivity meter. The 0.4 g leaves were weighed and put into a 50 mL centrifuge tube, 40 mL of deionized water was added, and vacuum was pumped until the leaves were completely submerged. The electrical conductivity of the extract solution (R1) was measured by the immersion method [24], heated in a boiling water bath for 30 min, and then the electrical conductivity of the extract solution (R2) was measured after cooling, which was repeated three times.

REC = R1/R2 × 100%(6)

Antioxidant enzymes (SOD, POD) activity measurement: SOD activity was determined by the nitrogen blue tetrazolium colorimetric method (NBT method), while POD activity was determined by the guaiacol method [25].

Osmoregulatory substances (Pro, SS, SP) measurement: proline (Pro) content was analyzed with the acid ninhydrin method [26], soluble sugars (SS) were measured using the thiobarbituric acid method [1], and soluble proteins (SP) were quantified using the Coomassie brilliant Blue G-250 method [27].

Quantification of plant hormones (ABA, ZR, IAA, GA3): the contents of abscisic acid (ABA), zeatin riboside (ZR), indole-3-acetic acid (IAA), and gibberellic acid (GA3) were measured using enzyme-linked immunosorbent assay (ELISA) according to the manufacturer’s instructions (Jiangsu Jingmei Biological Technology Co., Ltd., Yancheng, China).

Analysis of macronutrients and trace elements in roots: the concentrations of macronutrients (Na, K, Ca, Mg, and P) and trace elements (Fe, Mn, Zn, and Cu) in the roots were determined by inductively coupled plasma emission spectrometry (iCAP Pro XP, Thermo Fisher, Waltham, MA, USA) [17].

### 2.3. Data Statistics and Analysis

A two-way ANOVA was employed to rigorously evaluate the impact and statistical significance of stress treatment, AMF, and their interaction on each index. To discern differences among distinct treatments, a one-way ANOVA was conducted, with Duncan’s multiple range test serving as the post-hoc analysis tool to identify statistically significant differences at a threshold of *p* < 0.05. Prior to statistical analysis, the homogeneity of variance was verified for the dataset; any data that failed this criterion were appropriately transformed to adhere to the required standard. A significance level of *p* < 0.05 was established as the threshold for statistical significance. All data presented in charts and tables are presented in their raw form, accompanied by the mean ± standard error (mean ± SE) for enhanced clarity and reproducibility.

GraphPad Prism 8.0 and Excel 2019 were used to draw tables and data, Adobe Photoshop 2023 software was used for picture editing, and IBM SPSS Statistics 26 software was used for significance analysis of physiological indicators. Origin 2021 software was used for principal component analysis to extract principal components whose cumulative variance contribution rate was greater than 85% for reduction and simplification. The membership function method was further applied to standardize the indicators obtained from the analysis of seedlings with 9 different treatments on each principal component Equations (7) and (8), with the variance contribution rate of the principal component as the weight. Calculate the weight Wj (9) of each extracted principal component, and calculate the D value:

U(Xij1) = (Xij − Xjmin)/(Xjmax − Xjmin) (7)

U(Xij2) = 1 − (Xij − Xjmin)/(Xjmax − Xjmin)(8)

D = Σ(Xij × Wj)(9)

where Xij represents the jth membership function value of the ith treatment, Xij is the content of the jth indicator of the ith treatment, and Xjmax and Xjmin are the maximum and minimum values of the jth indicator among all treatments. μ(Xij1) and μ(Xij2) represent the conversion values of positive and negative indicators, respectively.

## 3. Results and Analysis

### 3.1. Effect of Saline–Alkaline Stress on AMF Colonization in the Roots of Black Wolfberry

**Figure 2:**
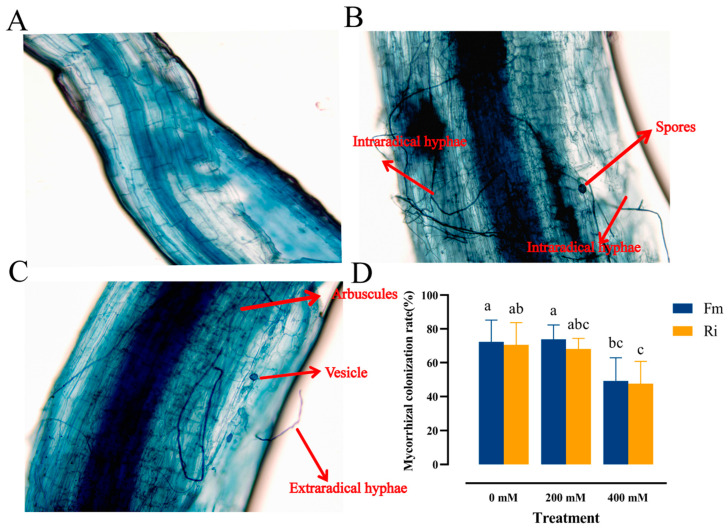
The root colonization of AMF under the microscope (400×) and their effective colonization rate and spore number. (**A**) NM treatment group. (**B**) Fm treatment group. (**C**) Ri treatment group. (**D**) Mycorrhizal colonization rate. The results are represented as the mean ± standard deviation of the three replicates. Different letters indicate differences between different treatments (*p* < 0.05).

A robust symbiotic partnership was established between the seedlings of *Lycium ruthenicum* and AMF fungi, leading to the development of vesicles, mycelia, and spore structures within the roots. Notably, the NM group exhibited no discernible signs of AMF colonization (Figure 2A), in stark contrast to the roots inoculated with Fm and Ri strains, which displayed significant colonization by mycelium and spores (Figure 2B,C). Notably, seedlings inoculated with Fm exhibited a superior colonization rate of 72.38% compared to those inoculated with Ri. Upon exposure to 200 mM salt–alkali stress, the infection rate of Fm-inoculated plants augmented, whereas it decreased in Ri-inoculated plants. When the concentration escalated to 400 mM, a marked decline in the affinity of both inoculated strains towards the roots of *Lycium ruthenicum* was observed. Nevertheless, the infection rate of Fm-inoculated plants remained relatively high at 49.19%, surpassing that of Ri-inoculated plants. This underscores the ability of both Fm and Ri to forge a beneficial symbiotic relationship with *Lycium ruthenicum*, with Fm emerging as an exceptional strain, demonstrating robust infection efficacy even under stressful conditions.

### 3.2. Effects of Diverse AMF on the Growth of Lycium ruthenicum under Saline–Alkaline Stress

Table 2 presents a comprehensive assessment of the influence of AMF on the root development of *Lycium ruthenicum* subjected to salt–alkali stress, along with a detailed two-way ANOVA analysis. Our findings reveal that neither AMF treatment alone nor the interplay between AMF and salt–alkali stress exerts a notable effect on SFW. Notably, as stress intensity escalates, the SFW of NM and Fm-inoculated plants undergoes marked suppression, with reductions of 58.03% and 43.90%, respectively, compared with the control group. Conversely, the change in plants inoculated with Ri was not significant. Regarding RFW, a significant interaction between salt–alkali stress × AMF emerges. Specifically, at a stress concentration of 400 mM, RFW declines by 61.89% and 43.86% in Fm- and Ri-inoculated plants, respectively, compared to the control. Intriguingly, at 0 mM stress, Fm-inoculated plants exhibit a 13.26% increase in RFW over their NM counterparts, whereas Ri-inoculated plants show no discernible change. Furthermore, RDW is profoundly influenced by both AMF and saline–alkaline stress. At 200 mM stress, Fm inoculation notably enhances RDW by 61.29% compared to NM, though this stimulatory effect diminishes at higher stress concentrations, indicating that, in contrast to Ri, Fm inoculation exerts a positive influence on RDW, and the promoting effect is weakened at high concentrations.

### 3.3. Effect of Diverse AMF on Photosynthetic Pigment Accumulation of Lycium ruthenicum under Saline–Alkaline Stress

**Figure 3:**
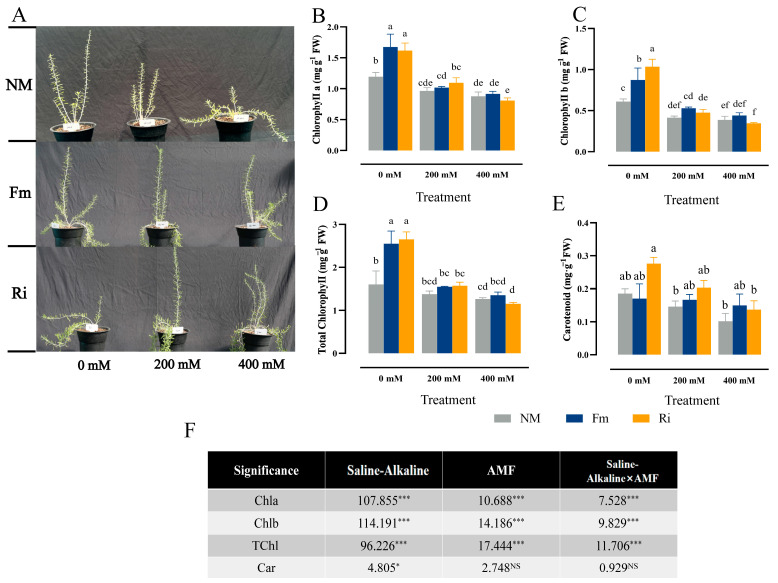
Changes in the photosynthetic pigments under saline–alkaline stress in leaves of seedlings. (**A**) morphology of seedlings, (**B**) Chlorophyll a (Chla) content, (**C**) chlorophyll b (Chlb) content, (**D**) TChl content, and (**E**) carotenoid (Car) contents in leaves. (**F**) The mean comparison using Duncan’s test (*p* < 0.05) was implemented to examine the differences among the treatments. Significant differences: * *p* < 0.05, *** *p* < 0.001, and NS, non-significant.

In comparison to NM0, Fm0 fostered above-ground growth, whereas Fm200 and Fm400 exhibited inhibiting effects. Conversely, prominently enhanced aboveground growth compared to NM400 (Figure 3A). Inoculation with Fm and Ri mitigated the chlorophyll content caused by stress. Specifically, at 0 mM stress concentration, compared with the Ri treatment group, Fm inoculation had a better effect on Chla promotion, but there was no significant difference between Fm0 and Ri0. Upon escalating stress to 400 mM, Chla levels in Fm-inoculated plants marginally surpassed those in NM, but there was no statistical difference (*p* < 0.05) (Figure 3B). The Fm0 group accumulated the most under the three treatments. At 0 mM treatment, Fm and Ri inoculations significantly promoted the accumulation, with respective increments of 69.63% and 43.34% compared with NM0. However, as stress intensity escalated, Chlb of the Fm treatment group was consistently higher than that of the Ri treatment group, underscoring a notable interaction between saline–alkaline stress × AMF type and Chlb content (Figure 3C,F). Similarly, Fm0 and Ri0 significantly enhanced total chlorophyll (TChl) accumulation by 59.36% and 65.77%, respectively, compared to NM0. However, this promotional effect diminished with increasing stress, eliminating significant differences among treatments. Notably, both saline–alkaline stress×AMF factors had distinct impacts on TChl levels (Figure 3D,F). In contrast to the aforementioned trends, both Fm and Ri treatments at 200 mM and 400 mM stress concentrations elevated Car content in *Lycium ruthenicum*, but there was no significant difference (*p* < 0.05). Notably, under 400 mM stress, Fm-inoculated plants exhibited a higher Car content than those treated with NM and Ri (Figure 3E).

### 3.4. Effect of Diverse AMF on Membrane Permeability of Lycium ruthenicum under Saline–Alkaline Stress

**Figure 4:**
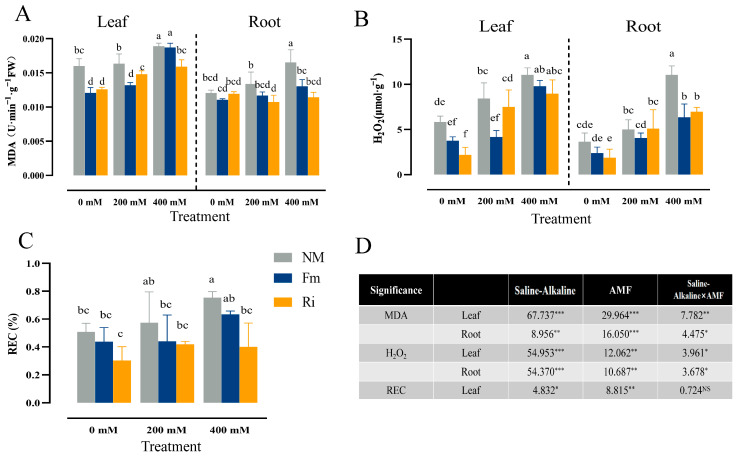
Changes in the lipid peroxidation and ROS in shoots and roots under different saline–alkaline conditions. (**A**) MDA, (**B**) H_2_O_2_, (**C**) REC contents of seedlings. The data are shown as the means ±SE from three biological replicates (*n* = 3). Letters in the upper part of the bar graph indicate significant differences between treatments. (**D**) The mean comparison using Duncan’s test (*p* < 0.05) was implemented for examining the differences among the treatments. Significant differences: * *p* < 0.05, ** *p* < 0.01, *** *p* < 0.001 and NS, non-significant.

Our investigation delved into the intricate interplay between salt–alkali stress and the lipid peroxidation processes in the leaves and roots of *Lycium ruthenicum*. Specifically, under unstressed conditions (0 mM), the inoculation of either Fm or Ri AMF led to a marked decline in malondialdehyde (MDA) content within the leaves, with reductions of 24.50% and 21.23%, respectively, compared to the NM group, but there was no significant difference in the inhibitory effect between the two strains. However, as stress intensity escalated to 200 mM, a discernible difference emerged, with Fm-inoculated plants exhibiting a significantly lower MDA content of 18.34% compared to their Ri-inoculated counterparts. When the stress concentration reached 400 mM, both Fm and Ri demonstrated substantial inhibition of MDA accumulation in roots, with respective decreases of 21.09% and 30.81%, Compared with the Fm treatment group, the MDA content of plants inoculated with Ri was lower (Figure 4A).

In contrast to the MDA trends, At 0 mM, solely Ri inoculation effectively mitigated H_2_O_2_ accumulation in leaves. Compared with the Ri treatment group, inoculation with Fm at 200 mM stress can inhibit H_2_O_2_ accumulation more effectively, achieving a significant 44.44% reduction compared to Ri-inoculated plants. Nevertheless, at the highest stress level, all treatments, including NM, Fm, and Ri, failed to elicit significant differences in H_2_O_2_ content in leaves. Conversely, in roots subjected to 400 mM stress, both AMF treatments significantly reduced H_2_O_2_ levels, though no significant distinction was observed between Fm and Ri in their efficacy (Figure 4B). Upon both 0 mM and 200 mM treatments, the leaf REC levels in plants inoculated with AMF exhibited a noticeable decline in comparison to their non-mycorrhizal (NM) counterparts, albeit the differences among the three groups were statistically insignificant. However, when the stress concentration escalated to 400 mM, plants inoculated with Ri significantly inhibited leaf REC compared with those treated with Fm (Figure 4C).

A two-factor analysis of variance revealed that salt–alkali stress imposed significant impacts on concentrations of MDA and H_2_O_2_ in both leaves and roots. Additionally, AMF inoculation had pronounced effects on MDA, H_2_O_2_, as well as REC levels. Notably, the interplay between salt–alkali stress and AMF did not significantly influence REC levels (Figure 4D).

### 3.5. Effects of Diverse AMF on Osmotic Regulation and Antioxidant Activity of Lycium ruthenicum under Saline–Alkaline Stress

**Figure 5:**
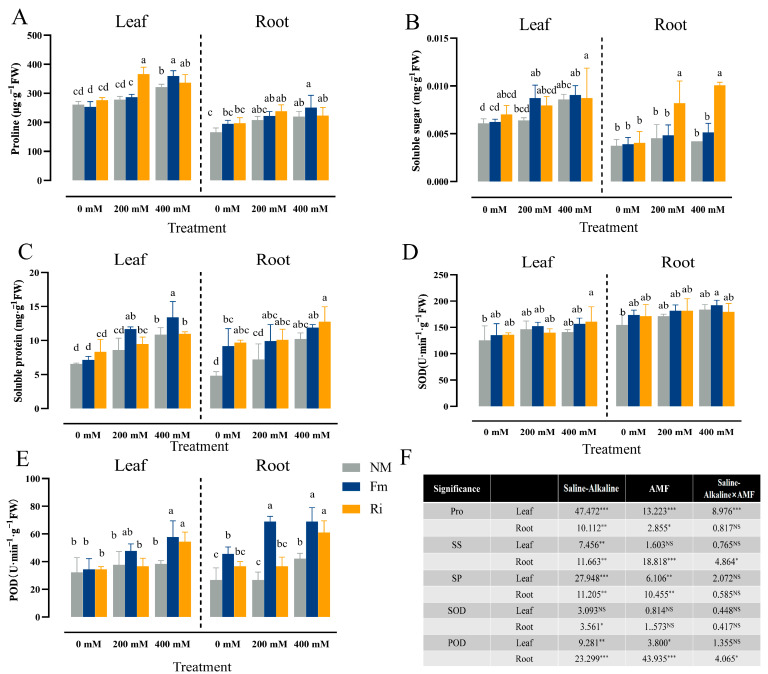
Changes in the content of osmolytes and non-enzymatic antioxidant compounds in leaves and roots under saline–alkaline conditions. (**A**) Pro, (**B**) SS, (**C**) SP, (**D**) SOD activity, (**E**) POD activity. The data are shown as the means ± SE from three biological replicates (*n* = 3). Letters in the upper part of the bar graph indicate significant differences between cultivars and treatments. (**F**) The mean comparison using Duncan’s test (*p* < 0.05) was implemented for examining the differences among the treatments. Significant differences: * *p* < 0.05, ** *p* < 0.01, *** *p* < 0.001 and NS, non-significant.

In this experiment, at 200 mM stress concentration, the accumulation of Pro was significantly higher than that of Fm200, which increased by 27.75% compared with Fm200. As the stress concentration intensified, the Pro content in the Fm400 treatment group marginally surpassed that of Ri400, registering a 12.03% elevation over NM400. Across stress levels of 0, 200, and 400 mM, the Fm treatment consistently exhibited higher Pro content than NM and Ri, albeit the differences were statistically insignificant (Figure 5A).

SS content in leaves exhibited an upward trend with escalating stress concentrations, yet no discernible statistical variation was observed among the treatment groups. Notably, Ri inoculation under 200 mM stress significantly enhanced SS accumulation in roots, which was significantly higher than that in the NM200 and Fm200 treatment groups, with Ri200 showcasing a 69.35% increase over Fm200 (Figure 5B). Furthermore, with the increase in stress concentration, there was a significant difference in SP accumulation in the leaves of the Fm treatment group and the Ri treatment group: the Fm treatment group significantly increased under 400 mM treatment, whereas the Ri400 group showed no significant change. In addition, AMF inoculation under 0 mM effectively promoted the accumulation of SP in roots, and the accumulation of Ri0 was greater than that of Fm0. Compared with NM0, Fm0 and Ri0 increased by 88.76% and 99.82%, respectively, albeit the difference between Fm0 and Ri0 was statistically insignificant (Figure 5C).

As stress concentration intensified, SOD activity in both leaves and roots exhibited an upward trend, albeit the magnitude of this change remained statistically insignificant (Figure 5D). Notably, at a concentration of 400 mM, POD activity in leaves underwent a marked enhancement upon inoculation with Fm and Ri, with the Fm400 treatment displaying a marginal superiority over Ri400. Intriguingly, inoculating Fm at 0 mM effectively promoted POD activity in roots, and the accumulation was higher than that in the Ri treatment group, surpassing Ri inoculation by a notable 24.24%. This trend persisted, with POD activity in roots continuing to rise significantly alongside escalating stress levels. Specifically, within the Fm200 treatment group, POD activity surpassed that of both NM200 and Ri200, whereas Ri-inoculated plants exhibited a substantial surge in POD activity at 400 mM, registering a significant 44.74% increase over NM400 (Figure 5E).

A two-way ANOVA analysis revealed the interaction of AMF and AMF × salt–alkali stress had a significant effect on SS content in leaves, while exhibiting no discernible effect on Pro content in roots. Concurrently, both salt–alkali stress emerged as potent factors modulating POD activity in both leaf and root tissues. Conversely, AMF exerted a significant influence solely on POD activity within root tissue (Figure 5F).

### 3.6. Effect of Diverse AMF on Endogenous Hormones of Lycium ruthenicum under Saline–Alkaline Stress

**Figure 6:**
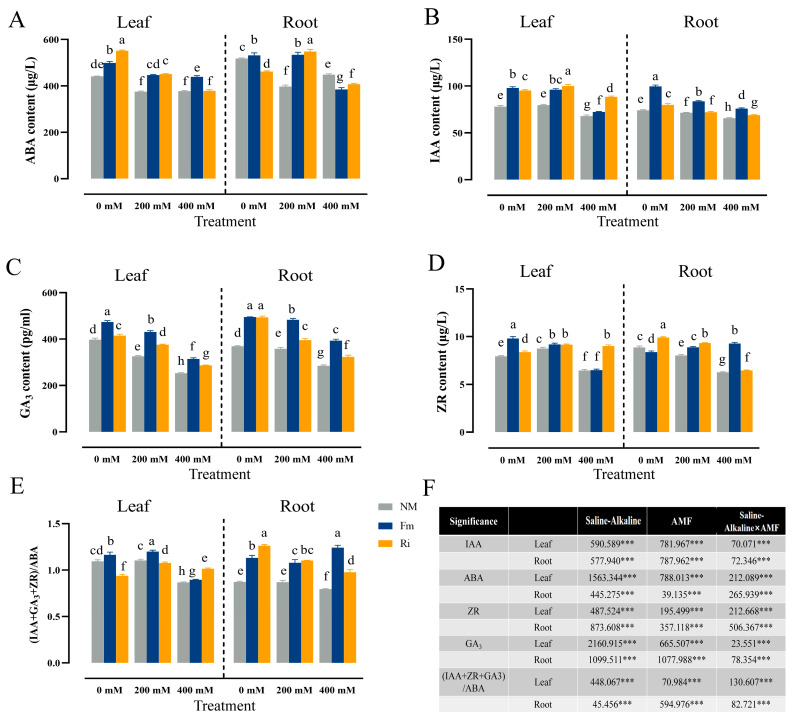
Changes in the content of endogenous hormones contents of seedlings. (**A**) ABA contents; (**B**) IAA contents; (**C**) GA_3_ contents; (**D**) ZR contents; (**E**) (IAA + GA_3_ + ZR)/ABA value. The data are shown as the means ± SE from three biological replicates (*n* = 3). Letters in the upper part of the bar graph indicate significant differences between cultivars and treatments. (**F**) The mean comparison using Duncan’s test (*p* < 0.05) was implemented for examining the differences among the treatments. Significant differences: *** *p* < 0.001.

The inoculation of AMF facilitated the accumulation of ABA in leaves, with Ri-inoculated plants exhibiting an initial advantage at 0 mM salt–alkali concentration, boasting a 10.66% higher ABA content than Fi0. As stress escalated to 200 mM, both AMF strains effectively elevated ABA levels in leaves, albeit without significant inter-strain differences. Notably, ABA content in Fm inoculated with 400 mM treatment was significantly higher than that in the Ri treatment group, which increased by 15.97% compared with Ri400, indicating its positive regulatory effect on ABA. In roots, at 0 mM stress, Fm inoculation augmented ABA accumulation, whereas Ri0 treatment led to a 10.83% decrease compared to NM0. At 200 mM, a similar trend emerged in both tissues, with Ri inoculation significantly enhancing ABA levels. However, with the continuous increase in stress concentration, AMF inoculation inhibited its accumulation, and the decrease in Fm inoculation was greater than that of Ri, which was 14.24% and 9.17% lower than NM, respectively (Figure 6A).

Saline–alkaline stress had a minimal impact on IAA content in leaves and roots, yet AMF inoculation emerged as a potent stimulant for IAA accumulation in leaves. Moreover, the Ri treatment group accumulated a higher IAA content than Fm. Specifically, under Fm200 and Ri200 treatments, IAA content significantly surged by 20.78% and 25.60%, respectively, compared to NM200. At 400 mM, Ri inoculation exhibited a more pronounced effect, elevating IAA levels by 22.18% over Fm400. Furthermore, across all stress concentrations (0 mM, 200 mM, and 400 mM), Fm inoculation significantly increased IAA content in roots, maintaining the highest concentrations and recording significant increases of 24.56%, 16.28%, and 10.11% over Ri0, Ri200, and Ri400, respectively (Figure 6B).

Salt–alkali stress impeded GA3 accumulation in leaves, yet AMF inoculation conspicuously augmented GA3 content across all salt concentrations. Specifically, leaves treated with Fm400 and Ri400 exhibited a 24.22% and 13.31% surge in GA3 content, respectively, over NM400. Notably, the Fm treatment group consistently demonstrated higher GA3 levels than Ri at comparable stress levels. Similarly, at 0 mM, 200 mM, and 400 mM concentrations, Fm0, Fm200, and Fm400 significantly increased GA3 content by 14.19%, 14.52%, and 9.63%, respectively, compared to their Ri counterparts. In unstressed roots (0 mM), both Fm and Ri fostered GA3 accumulation, albeit without significant differences. However, as stress intensified, GA3 content declined, with Fm inoculation proving more effective than Ri in mitigating this decline. Remarkably, Fi200 and Fi400 treatments boosted leaf GA3 content by 22.21% and 21.67%, respectively, over Ri200 and Ri400 (Figure 6C).

At 0 mM and 200 mM concentrations, both AMF strains effectively elevated ZR content in leaves, with Fm0 treatment yielding the highest ZR levels, a significant 17.36% increase over Ri0. Conversely, at 400 mM, the Ri treatment surpassed Fm400, registering a remarkable 39.09% increase in ZR content. In roots, ZR content in the Fm400 treatment group was significantly higher than that in other groups, and showed a rising trend versus Fm200, achieving a 44.10% increase over Ri400 (Figure 6D).

Under saline–alkaline stress, the ratio of IAA + ZR + GA3 to ABA generally declined. Notably, Fm inoculation at 0 mM significantly bolstered this ratio in leaves, while Ri400 surpassed Fi400 by 13.32% at 400 mM. In roots, both Fm and Ri significantly promoted the ratio, with Fm peaking at 400 mM treatment, achieving a 27.15% higher than Ri400 (Figure 6E).

A two-way ANOVA analysis revealed that the interaction of salt–alkali stress, AMF, and AMF × saline–alkaline stress had significant effects on ABA, IAA, GA3, and ZR contents (Figure 6F).

### 3.7. Effect of Diverse AMF under Salt and Alkali Stress on Mineral Element Content of Root of Lycium ruthenicum under Saline–Alkaline Stress

#### 3.7.1. Effect of Diverse AMF under Salt and Alkali Stress on the Content of Macroelements in the Root of *Lycium ruthenicum*

**Figure 7:**
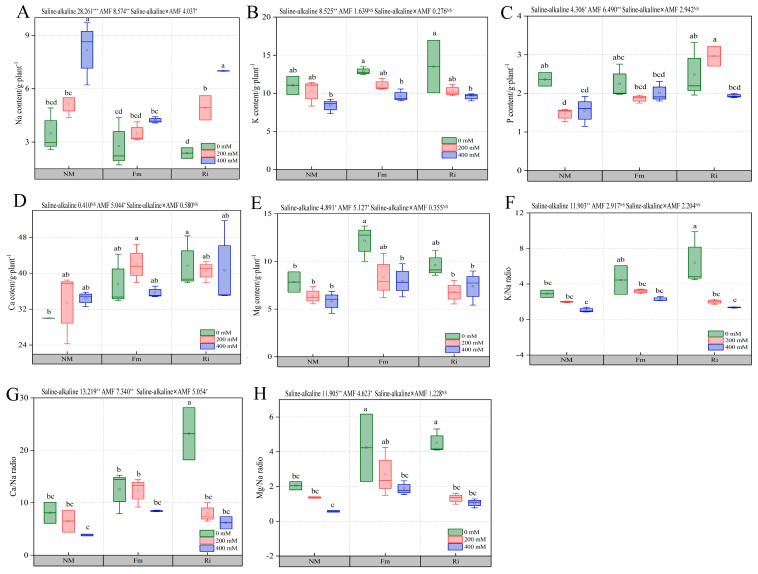
Changes in the content of macroelement in Wolfberry root system. (**A**) Na, (**B**) K, (**C**) P, (**D**) Ca, (**E**) Mg, (**F**) K/Na, (**G**) Ca/Na, (**H**) Mg/Na. The data are shown as the means ± SE from three biological replicates (*n* = 3). Letters in the upper part of the bar graph indicate significant differences between cultivars and treatments. (**F**) The mean comparison using Duncan’s test (*p* < 0.05) was implemented for examining the differences among the treatments of significant differences: * *p* < 0.05, ** *p* < 0.01, *** *p* < 0.001 and NS, non-significant.

As the concentration of saline–alkaline stress increased, the overall Na content in the NM treatment, exhibited a pronounced upward trend, whereas the accumulation of Na was mitigated through inoculation with Fm and Ri. Notably, at stress concentrations of 0 mM and 200 mM, no discernible variation in AMF inoculation effects was observed among the various treatments. However, at the elevated concentration of 400 mM, compared with other treatments, only the Fm treatment group significantly inhibited Na content in leaves, achieving a significant 39.65% reduction compared to Ri400 (Figure 7A). Analogously, salt–alkali stress inhibited the K content, yet inoculation with both Fm and Ri modestly enhanced K accumulation, albeit the differences were statistically insignificant (Figure 7B).

Furthermore, AMF inoculation proved beneficial in mitigating the decline of P content in seedling roots. Under non-stress conditions, no significant distinction was noted between Fm and Ri treatments. At 200 mM stress, while Fm and NM treatments were comparable, Ri-inoculated plants exhibited a notable promotion of P accumulation, surpassing Fm200 by 59.21%. Even under the severe stress of 400 mM, seedlings inoculated with Fm and Ri displayed a trend towards increased P content, albeit this increase was not statistically significant (Figure 7C).

AMF inoculation promoted Ca accumulation in roots to a certain extent, and the increase in the Ri treatment group was greater: Ri0 treatment prominently elevated Ca content in comparison to NM0, while Fm0 displayed a marginally higher level, albeit without statistical significance. Notably, under 200 mM and 400 mM stress conditions, no significant alterations were observed in Ca content among NM, Fm, and Ri treatments (Figure 7D). Regarding Mg content in the root of *Lycium ruthenicum*, saline–alkaline stress imposed inhibitory effects. Under non-stress conditions, Fm inoculation bolstered Mg content by 55.20% compared to NM0, yet it did not significantly differ from Ri0. With the increase in stress concentration, Mg content in both Fm and Ri treatments gradually increased, but not significantly (Figure 7E).

Under non-stress conditions, inoculation significantly enhanced the root K/Na ratio by 12.06% in comparison to NM0, though this elevation was not significantly different from that achieved by Fm0. As stress levels rose, no discernible variation was observed in the K/Na ratio between NM and AMF plants (Figure 7F). Moreover, Ri inoculation significantly augmented Ca/Na under non-stress conditions, with Ri0 yielding 186.44% and 84.83% higher values than NM0 and Fm0 treatments, respectively. However, at 200 mM and 400 mM stress, no significant differences were detected between the NM, Fm, and Ri treatments (Figure 7G). The trend in Mg/Na ratio paralleled that of Ca/Na, as both Fm0 and Ri0 treatments significantly increased Mg/Na in roots by 107.00% and 121.13%, respectively, compared to NM0, with no significant difference between them (Figure 7H).

#### 3.7.2. Effect of Diverse AMF under Salt and Alkali Stress on Trace Element Content of Root System of *Lycium ruthenicum* under Saline–Alkaline Stress

**Figure 8:**
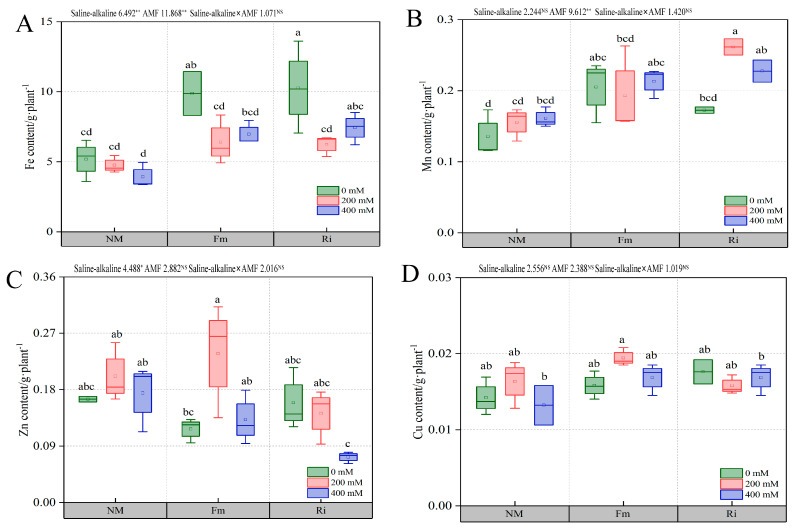
Changes in the content of microelement in wolfberry root system. (**A**) Fe, (**B**) Mn, (**C**) Cu, (**D**) Zn. The data are shown as the means ± SE from three biological replicates (*n* = 3). Letters in the upper part of the bar graph indicate significant differences between cultivars and treatments. The mean comparison using Duncan’s test (*p* < 0.05) was implemented for examining the differences among the treatments of significant differences: * *p* < 0.05, ** *p* < 0.01, and NS, non-significant.

Under non-stressful conditions, the inoculation of Fm and Ri significantly increased Fe content, yet the difference between Fm0 and Ri0 treatments remained statistically insignificant. Specifically, Fe content under Fm0 and Ri0 treatments escalated by 90.64% and 98.51%, respectively, in comparison to NM0. Notably, Ri400 treatment markedly fostered Fe accumulation when contrasted with NM400, although no significant distinction was discernible between Ri400 and Fm400 (Figure 8A).

Mn content in the Fm treatment group peaked under 0 mM treatment, exhibiting a 51.48% increase over NM0, without a statistically significant divergence from Ri0. However, under 200 mM stress, Mn content in Ri treatment surpassed that of all other groups, achieving a 35.73% elevation compared to Fm200, whereas at 400 mM stress, no notable differences were observed among treatment groups (Figure 8B).

At 0 mM and 200 mM stress levels, Zn content remained relatively stable across the NM, Fm, and Ri treatment groups. However, at a concentration of 400 mM, Zn content in Ri-inoculated plants significantly decreased, exhibiting a 45.20% reduction compared to Fm400 (Figure 8C). Furthermore, Cu content remained largely unchanged across all treatment groups under varying stress conditions (Figure 8D).

### 3.8. Evaluation of Correlation between Mineral Elements and Physiological Indices

**Figure 9:**
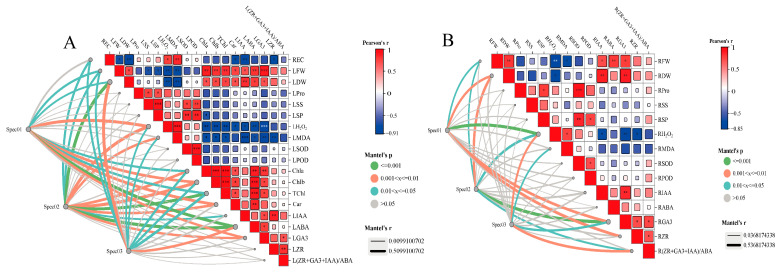
The regulatory relationship between physiological traits and mineral elements. (**A**) Based on Mantel test analysis, the correlations between leaf physiological traits and large and trace elements were revealed. (**B**) Based on Mantel test analysis, correlations between root physiological traits of Wolfberry and macroelements and trace elements were revealed. Spec01 represents the Na content under Nm, Fm and Ri treatment. Spec02 represents the accumulation of macroelements including K, K/Na, P, Ca, Mg, Ca/Na, Mg/Na under Nm, Fm, Ri treatment. Spec03 represents the accumulation of trace elements including Fe, Mn, Zn, and Cu under the treatment of Nm, Fm, and Ri. The mean comparison using Duncan’s test (*p* < 0.05) was implemented for examining the differ-ences among the treatments of significant differences: * *p* < 0.05, ** *p* < 0.01, *** *p* < 0.001.

In order to further explore the correlation between mineral element accumulation and the physiological traits of seedlings, the above factors were evaluated by a Mantel test. Na accumulation was significantly correlated with LABA content (*p* < 0.001) and LH_2_O_2_, Car, and Chla content (*p* < 0.01). The accumulation of macroelements such as K, Ca, and Mg exhibited a notable association with leaf physiological indices, demonstrating a highly significant correlation with TChl, LH_2_O_2_, LMDA, LABA, and LGA3 contents (*p* < 0.01). Interestingly, the trace elements Fe, Mn, Zn, and Cu also displayed a remarkable correlation with LGA3 and LABA levels (*p* < 0.001) (Figure 9A).

In the root system, the accumulation of Na was significantly correlated with the accumulation of RDW, RZR, and RGA3 (*p* < 0.01). Similarly, the accumulation of K, Ca, and Mg was intimately linked to RGA3 accumulation (*p* < 0.001), while RDW and RFW also exhibited a significant correlation (*p* < 0.05). Notably, the accumulation of trace elements Fe, Mn, Zn, and Cu was found to be significantly correlated with the hormonal balance R(IAA + GA3 + ZR)/ABA (*p* < 0.01) (Figure 9B).

### 3.9. Comprehensive Evaluation of Lycium ruthenicum under Saline–Alkaline Stress

Employing color intensity to generate a cluster heat map, the performance of 45 physiological indicators under 9 treatments can be visualized. Utilizing Pearson correlation as the cluster kernel, these indicators were systematically categorized into four distinct clusters. Cluster a included Zn and Cu, while Cluster b encompasses a broad range of parameters, including IAA, ABA, ZR, and GA3 contents in both leaves and roots, along with all mineral elements except Na, P, Ca, Mn, LDW, RDW, LFW, RFW, and chlorophyll levels. Cluster c specifically includes the accumulation of H_2_O_2_, MDA, and Na in leaf and root REC. Cluster d compiles a significant array of elements (P, Ca) and trace elements (Mn) in leaves and roots, along with SOD, POD activities, and osmotic regulators (Pro, SS, SP).

Color intensity variations within these clusters reveal intriguing trends. Notably, under non-stress conditions, the LABA in cluster b treated with Ri is elevated. However, as stress concentrations escalate, the contents of LABA, RIAA, LGA3, and RGA3 in Fm-treated samples surpass those of the Ri-treated and NM groups, highlighting the beneficial effects of Fm treatment. Additionally, Ri-treated samples exhibit higher K/Na ratios and Chlb contents, indicative of enhanced salt tolerance. In Cluster c, the accumulation of RMDA, LH_2_O_2_, and Na intensifies with increasing stress, yet the Fm-treated group exhibits lower levels compared to Ri, suggesting reduced membrane damage. Cluster d underscores the superiority of Fm-inoculated roots and leaves, displaying heightened antioxidant enzyme activities and osmoregulatory substance levels that escalate markedly with stress intensity, indicating a more robust stress adaptation strategy.

**Figure 10:**
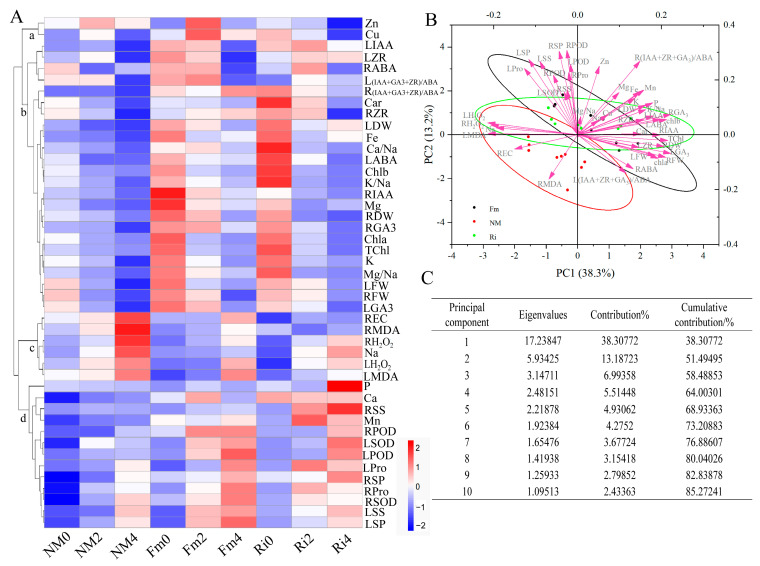
Results of evaluated parameters in seedlings under AMF and saline–alkaline stress conditions. (**A**) Cluster heat maps of physiological indexes of seedlings in each treatment group, (**B**) principal component analysis (PCA) results of 45 evaluation index of seedlings, (**C**) variance and contribution rate of each principal component.

To delve deeper into the disparities in the effects of two inoculation methods under salt stress, principal component analysis (PCA) was performed on the physiological indexes of seedlings. Adhering to the criteria of an initial eigenvalue > 1 and a cumulative contribution rate exceeding 85%, 10 principal components (PCs) are calculated, and the cumulative contribution rate is 85.3%. This comprehensive coverage effectively encapsulates the majority of the measured index data, providing a holistic view of the treatment impacts on the seedlings (Figure 10A). Notably, PC1 contributes the lion’s share of this information (38.3%), followed by PC2 with 13.2%.

Specifically, PC1 encompasses a diverse array of factors, including LABA, LIAA, RIAA, RGA3, K/Na ratio, LH_2_O_2_, RH_2_O_2_, LMDA, and Na content, while PC2 focuses on LPOD, RPOD, LSP, RSP, LSS, and RMDA activities and levels (Figure 10B). These findings underscore the significance of endogenous hormones, Na content, and membrane permeability indices as pivotal determinants in assessing the differential regulation of salt–alkali tolerance under two conditions of inoculation.

To overcome the constraints of single-index analysis and gain a comprehensive understanding of the merits and drawbacks of each AMF treatment, the membership function formula was employed (Table 3) to calculate the D values of the comprehensive indices of the NM, Fm, and Ri groups. This enabled us to conduct a comprehensive ranking of the salt tolerance capabilities. By determining the contribution rate (D value) of each comprehensive index, we comprehensively ranked the tolerance of each treatment. The average D values for NM, Fm, and Ri were 0.343, 0.712, and 0.622, respectively, conclusively demonstrating that the Fm treatment group exhibited superior salt–alkali tolerance compared to the Ri treatment group, with both significantly outperforming the NM group.

## 4. Discussion

Saline–alkaline stress imposes multifaceted constraints on plant growth and development, yet colonization by AMF acts as a catalyst, bolstering plant growth and physiological vitality, thereby significantly reinforcing the host plant’s resilience against such adverse conditions [28]. Liu [29] identified Ningxia’s Lycium berry rhizosphere and revealed ten distinct AMF spore species based on their morphological characteristics. Xiao [30] illuminated the dominance of Fm as the primary AMF in symbiosis with Lycium berries. In addition, Wei [31] found that Ri’s capacity to upregulate potassium ion channel gene expression enhanced drought tolerance. This comprehensive study delves into the comparative analysis of growth indices, mineral composition, endogenous hormones, and other vital physiological markers in *Lycium ruthenicum* inoculated with Fm and Ri under salt–alkali stress. The objective is to pinpoint the preeminent strain capable of bolstering the plant’s salt tolerance. Our experiment affirms that both Ri and Fm effectively colonize the root system of *Lycium ruthenicum*, fostering a mutually beneficial symbiosis. However, under intense saline–alkaline stress, the colonization rate of mycorrhiza diminishes, hinting at the inhibitory effect of high pH on AMF spore germination, ultimately diminishing arbuscule and vesicle formation, and constraining the extent of mycorrhiza infection [32]. Under non-stress conditions, Fm and Ri inoculations stimulate the accumulation of RFW (Table 2), suggesting that AM fungi mitigate salt–alkali stress by intricately reshaping root structure. This includes fostering longer taproots and shorter lateral roots, a phenomenon congruent with observations in cabbage [33]. As stress intensity escalated, compared with the Ri treatment group, Fm inoculation promoted RDW accumulation, hinting at a potential correlation between K element abundance and RDW. This implies that Fm inoculation might regulate the development of K-absorbing roots, echoing findings in *Lycium ruthenicum* [34].

Chlorophyll, the indispensable photosensitive catalyst of photosynthesis, serves as a pivotal indicator, mirroring the vigor and efficiency of photosynthetic activity [35]. Our study reveals that saline–alkaline stress exerts a detrimental influence on the photosynthetic apparatus of seedlings, markedly diminishing the concentrations of Chla, Chlb, and Car (Figure 3B,C,E). This decline is likely attributed to elevated ROS levels, which trigger chlorophyll degradation and chloroplast impairment, echoing similar observations made in cotton [12]. Intriguingly, compared with Fm-inoculated, Chlb content was significantly increased in Ri-inoculated, and P accumulation continued to increase under non-stress conditions (Figure 9A). This suggests that Ri inoculation might facilitate the expansion of the mycelial network, thereby enhancing the root’s phosphorus uptake capacity. As phosphorus is a fundamental nutrient crucial for chlorophyll synthesis, it thus promotes the production of chlorophyll and enhances photosynthetic capacity [36]. To delve deeper into the regulatory influence of arbuscular mycorrhizal fungi (AMF) on ROS scavenging, MDA and H_2_O_2_ levels were determined. Our results indicated a notable downward trend in MDA content among seedlings inoculated with AMF (Figure 4A,B), aligning with previous observations in cotton [37]. This decrease likely stems from AMF’s activation of robust antioxidant phosphate metabolism pathways, effectively mitigating membrane oxidative damage resulting from saline–alkaline stress, as demonstrated by *Populus×xiaohei* [38]. Moreover, research has revealed that Fm inoculation significantly upregulates antioxidant enzyme genes *AtAPX3* and *AtAPX4* in alfalfa, mitigating the inhibitory effects of soil pH on antioxidant enzyme activity [20]. This study also confirmed this phenomenon physiologically: as the intensity of saline–alkaline stress increased, SOD and POD activities remained largely unchanged in the NM treatment, whereas AMF inoculation, notably with Fm, substantially elevated POD activity, surpassing the enhancement observed with Ri (Figure 5D,E). This underscores AMF’s effectiveness in alleviating stress-induced membrane oxidative damage by concurrently activating both enzymatic and non-enzymatic antioxidant defense mechanisms within plants [7]. In general, AMF showed a certain degree of adaptability to saline–alkaline stress, while Fm showed a better ability to scavenge ROS than Ri, which reduced the damage to seedlings.

Under saline–alkaline stress, the biosynthesis and accumulation of small molecule osmolytes, including Pro, SS, and SP, play pivotal roles in enhancing cellular osmotic pressure, lowering water potential, and ultimately mitigating stress-induced damage in plants [39]. Our study revealed that the Ri treatment group significantly bolstered the accumulation of Pro and SS in both leaves and roots under high-concentration stress (Figure 5A,B). This suggests that Ri inoculation effectively improves the hydraulic properties of the host plant, functioning as a crucial mediator in enhancing the salt tolerance of *Lycium ruthenicum*, which is consistent with tomato [40]. Simultaneously, Fm inoculation significantly promoted the accumulation of SP in leaves at high stress concentrations (Figure 5C), indicating that Fm treatment could inhibit the decrease in SP content in leaves under high salt–alkali stress and promote the production of new proteins to resist adversity [41].

Saline–alkaline stress significantly perturbs the delicate hormonal equilibrium within plants, with ABA playing a pivotal role in regulating stomatal aperture to minimize water loss and facilitating the establishment of AMF symbiosis [42]. In this study, Fm inoculation significantly promoted ABA content in leaves and roots compared with Ri at 200 mM stress concentration (Figure 6A). Notably, a strong positive correlation emerged between Na^+^ accumulation and ABA content, suggesting that upon Na^+^ influx into the root system of *Lycium ruthenicum*, the Fm-treated group might mediate ABA biosynthesis, thereby stimulating Na^+^/H^+^ exchange activity and effectively expelling excess Na^+^ from the cells. This mechanism effectively alleviates ionic toxicity within the cytoplasm, aligning with findings in populus [14]. However, with the continuous increase in stress concentration, inoculation of both strains significantly reduced the content of ABA in roots, suggesting that AMF inoculation could reduce the accumulation of ABA in roots and thus increase salt tolerance. The results indicated that ABA in plants inoculated with Fm under salt–alkali stress mainly acts as stress signaling molecules to regulate the transduction of Na^+^ transporters, which is consistent with the results of strawberry [43].

Optimal concentrations of IAA vigorously stimulate plant root and leaf growth, exhibiting a strong positive correlation with biomass accumulation [44]. In this study, exposure to high saline–alkaline stress impeded IAA production in seedlings, whereas AMF inoculation effectively counteracted this effect by enhancing IAA accumulation in leaves, thereby mitigating stress-induced biomass depletion (Figure 6B). This finding aligns well with previous observations in cotton [42]. Moreover, Fm inoculation exhibited a more pronounced effect on boosting IAA synthesis in roots, suggesting its role in facilitating nutrient uptake in roots and contributing to an overall increase in biomass. AMF inoculation also significantly increased GA3 content in seedlings, likely by augmenting nutrient absorption efficiency in both leaves and roots (Figure 6C), as well as optimizing root architecture and function, thereby enabling robust physiological adaptation across different plant parts [45]. Notably, the stimulatory effect of Fm inoculation surpassed that of Ri, and this enhancement was significantly associated with the accumulation of K, Ca, and Mg. This implies that GA3 might act synergistically with Fm inoculation, which facilitates GA3 signal transduction under salt–alkali stress. This, in turn, regulates the expression of K ion transporters, enhancing osmotic regulation capabilities, which is congruent with the findings reported by Wang [46].

ZR, the most abundant cytokinin in plants, emerged as a key player in our study. In this study, with the increase in stress concentration, the promotion effect of inoculating Ri on ZR in leaves was more obvious (Figure 6D), indicating that inoculating Ri could regulate the accumulation of ZR and promote cell division and expansion in leaves of *Lycium ruthenicum* at high saline–alkaline concentrations, thus contributing to the maintenance or recovery of growth, which was consistent with the results of *Juglans nigra* [47]. However, the regulation patterns of the two AMFs in the root system differ: At low stress concentrations, the Ri-inoculated roots accumulate more ZR than the Fm-treated group, indicating that Ri-inoculated plants may utilize the synthesis of ZR in the root system to promote the synthesis and modification of cell wall pectin, enhancing cell wall stability and restricting the influx of Na^+^, which was studied in Creeping Bentgrass [48], As the stress increases, Fm-inoculated roots significantly promote the ZR content compared to Ri, combined with the correlation between RZR and Na^+^ (Figure 9B), indicating that Fm inoculation may activate the Na^+^ efflux mechanism, maintaining ion balance and thereby promoting the accumulation of RZR. The (IAA + GA3 + ZR)/ABA ratio serves as a pivotal indicator of the intricate balance between various endogenous hormones and their comprehensive influence on plant physiology. Our investigation revealed that AMF symbiosis significantly elevated this ratio in seedlings (Figure 6E), pointing to its capacity to augment cell elongation and division, thereby augmenting biomass accumulation and growth vitality. This is consistent with the results of the black walnut [47]. Furthermore, correlation analysis underscored the strong link between the (IAA + GA3 + ZR)/ABA ratio and trace element accumulation. Notably, Fm inoculation under high stress conditions further augmented this ratio, indicating that, compared with the Ri group, Fm could promote the comprehensive regulation of endogenous hormones, thereby enhancing trace element absorption and utilization. This is consistent with the results of citrus [49].

Under salt–alkali stress, plants undergo an intricate cascade of physiological adaptations, which prominently involve osmotic regulation through the strategic redistribution of inorganic ions. As salt accumulation surpasses a pivotal threshold, plants exhibit a tendency to sequester increased amounts of Na [50]. Plants undergo an intricate cascade of physiological adaptations, which prominently involve osmotic regulation through the strategic redistribution of inorganic ions. As salt accumulation surpasses a pivotal threshold, plants exhibit a tendency to sequester increased Na. This finding aligns with previous research on Casuarina glauca [51], further emphasizing that Fm inoculation demonstrated a more pronounced ability to diminish Na^+^ levels compared to Ri (Figure 7A), and this reduction was significantly associated with LH_2_O_2_ levels. This suggests that Fm-inoculated plants harness H_2_O_2_ signaling molecules to perceive stress, ignite the antioxidant defense machinery, and efficiently counteract the toxicity stemming from excessive Na, echoing findings in tomato research [52]. Given the competitive nature of Na and K for binding sites, which are vital for sustaining diverse metabolic processes, maintaining a high K/Na ratio is paramount to preventing cellular deterioration and nutritional deficiencies [39]. Notably, Ri inoculation conspicuously elevated the K/Na ratio in roots (Figure 7F), a phenomenon positively correlated with heightened levels of ZR and GA3 hormones. Consequently, under saline–alkaline stress, Ri inoculation safeguards a favorable K/Na balance by augmenting the production of ZR and GA3, thereby modulating the activity of ion uptake channels in roots. This finding aligns with previous studies on tomato [53]. Moreover, Mg, the cornerstone of plant chlorophyll molecules, is indispensable for photosynthesis regulation [54]. Our study highlights that salt–alkali stress impedes Mg content in seedling roots, whereas Fm inoculation counteracts this suppression (Figure 7E). This alleviation might stem from enhanced Mg absorption in roots and its subsequent translocation to shoots, ensuring continuous chlorophyll synthesis and preserving the nutritional integrity of aerial plant parts, thus mitigating ion imbalance caused by saline–alkaline stress, in line with observations in pistachio [28].

Trace elements, albeit in minimal proportions in plants, function as enzymatic or co-enzymatic components, displaying remarkable mobility and being indispensable for normal growth and development [55]. Fe stands out as a vital component for chlorophyll synthesis, predominantly residing in chloroplasts and playing a crucial part in the electron transport chain during photosynthesis. Our research demonstrates that AMF inoculation significantly enhances Fe accumulation (Figure 8A), thereby regulating chlorophyll synthesis, as evidenced in cucumber [56]. Zn, another essential trace element, is instrumental in promoting root growth [57]. Our findings revealed that under moderate stress concentrations, AMF inoculation promotes Zn content in roots, whereas elevated pH levels hinder Zn accumulation (Figure 8C). This suggests that, under adaptive stress conditions, AMF may release phenolic compounds or other substances that stimulate the uptake of mineral elements from the soil [2]. However, under severe saline–alkaline stress, this stimulatory effect is suppressed, impeding AMF’s ability to ensure adequate Zn supply, aligning with previous observations in tomato [57]. In contrast, Cu content in seedlings remains relatively low, and AMF inoculation exhibits negligible impact on its content, underscoring the plant’s minimal requirement for this element (Figure 8D). Collectively, these findings emphasize that nutrient elements in roots do not operate in isolation but instead interact synergistically to support plant growth. AMF inoculation facilitates a balanced supply of essential nutrients, meeting the growth demands of seedlings and safeguarding overall plant nutrition uptake [58]. Furthermore, simultaneous inoculation with Fm and Ri exhibits a preference for the absorption of mineral elements by plants, which determines the differences in the response mechanisms of different AMF strains to saline–alkaline stress [59].

At present, the comparison of stress tolerance between AMF-inoculated and NM plants has robustly demonstrated the promising application of mycorrhizalization in the holistic remediation of salt–alkali land for *Lycium ruthenicum* cultivation. Simultaneously, the distinct responses exhibited by Fm and Ri inoculations to salt–alkali stress offer invaluable insights for guiding the selection of optimal inoculation strategies in such soils. This understanding not only provides a solid scientific foundation but also technical support for the cultivation and management practices aimed at enhancing the productivity of *Lycium ruthenicum* in saline–alkaline environments.

## 5. Conclusions

Saline–alkaline stress significantly impedes the growth of *Lycium ruthenicum* seedlings by inhibiting chlorophyll synthesis, exacerbating membrane lipid peroxidation, and disrupting the delicate balance between endogenous hormones and nutrient elements. However, inoculation proves to be a potent strategy to bolster the seedlings’ resilience against such stresses. Specifically, Fm-inoculated plants elevate SOD and POD activities, thereby enhancing tolerance. Additionally, Fm regulates the absorption of element K, which is crucial for maintaining osmotic balance and fostering root growth. Conversely, Ri enhances light-harvesting capabilities primarily by promoting the uptake of element P and modulating ZR levels to mitigate the toxicity of Na. These findings may point to the possibility that inoculation by both fungi at once may be best. A comprehensive analysis utilizing the membership function underscores the superior performance of Fm inoculation in enhancing the salt–alkali tolerance of *Lycium ruthenicum*. Consequently, the mycorrhizalization of *Lycium ruthenicum* holds promising applications for the comprehensive remediation of salt–alkali soils, offering a viable solution for sustainable agriculture in challenging environments.

## Figures and Tables

**Table 1:** Physical and chemical properties of test substrates.

Index	pH	Dry Matter (g/L)	EC Value	Nitrate Nitrogen Content (g·kg^−1^)	Ammonium Nitrogen Content (g·kg^−1^)	Phosphorus Content (g·kg^−1^)
Matrix soil	6.0	55–75	2.0–4.0	70	50	140

**Table 2:** Combinative effects of AMF and saline–alkaline stress on the plant growth index of seedlings.

AMF	Saline–Alkaline (mM)	SFW (g)	RFW (g)	SDW (g)	RDW (g)
NM	0	12.39 ± 2.35 ab	17.14 ± 2.08 b	2.23 ± 0.96 bc	4.30 ± 0.26 bc
	200	8.67 ± 1.39 bc	10.11 ± 1.83 cd	1.60 ± 0.50 c	3.49 ± 0.59 cd
	400	5.20 ± 1.30 c	7.11 ± 2.89 d	1.31 ± 0.61 c	2.36 ± 1.00 cd
Fm	0	14.76 ± 4.83 a	19.76 ± 2.93 a	3.87 ± 0.38 a	7.49 ± 2.26 a
	200	9.70 ± 1.71 abc	16.31 ± 2.70 b	3.27 ± 1.25 ab	5.90 ± 1.01 ab
	400	8.28 ± 1.61 bc	7.53 ± 3.48 d	2.24 ± 0.01 bc	2.96 ± 1.58 cd
Ri	0	13.19 ± 2.03 ab	17.58 ± 1.92 b	4.32 ± 0.70 a	6.52 ± 1.29 a
	200	11.49 ± 1.335 ab	15.10 ± 1.04 bc	2.84 ± 1.02 abc	2.57 ± 0.57 cd
	400	8.20 ± 4.66 bc	9.87 ± 2.03 cd	3.24 ± 1.26 ab	1.79 ± 0.26 d
Significance					
Saline–alkaline		12.011 ***	51.605 ***	5.010 **	23.455 ***
AMF		1.976 NS	21.941 ***	10.845 ***	8.550 ***
Saline–alkaline×AMF		0.835 NS	9.936 ***	0.635 NS	0.088 NS

Notes: ** significant at *p* < 0.01 level; *** significant at *p* < 0.001; NS, not significant. Different letters in the same column represent significant differences by Ducan’s multiple test (*p* < 0.05), and there was no significant difference in the same letters.

**Table 3:** Analysis of the affiliation function of AMF colonized.

AMF	Saline–Alkaline (mM)	μ(1)	μ(2)	μ(3)	μ(4)	D Value	Average	Order
NM	0	0.61	0.00	0.37	0.39	0.47	0.34	3
	200	0.35	0.25	0.48	0.59	0.39		
	400	0.00	0.32	0.72	0.28	0.18		
Fm	0	0.98	0.59	0.41	0.37	0.85	0.71	1
	200	0.65	0.85	0.63	1.00	0.77		
	400	0.25	0.95	1.00	0.34	0.52		
Ri	0	1.00	0.70	0.75	0.00	0.90	0.62	2
	200	0.50	0.81	0.23	0.57	0.59		
	400	0.21	1.00	0.00	0.14	0.38		

## Data Availability

The raw data supporting the conclusions of this article will be made available by the authors on request.
